# Jasmonic acid signaling pathway repressor JAZ3 integrates light and temperature signaling in Arabidopsis

**DOI:** 10.1093/plphys/kiae164

**Published:** 2024-03-16

**Authors:** Kumari Billakurthi

**Affiliations:** Assistant Features Editor, Plant Physiology, American Society of Plant Biologists; Department of Plant Sciences, University of Cambridge, Downing Street, Cambridge CB2 3EA, UK

Light and temperature are fundamental environmental factors that profoundly influence plant growth and development. As sessile organisms, plants rely on their ability to perceive and integrate daily and seasonal variations in light and temperature to adjust their growth and development, enabling them to survive amid fluctuating weather conditions. Exposure to light orchestrates 2 distinct developmental programs: skotomorphogenesis in darkness and photomorphogenesis in the presence of light. Dark-grown seedlings exhibit etiolated phenotypes, characterized by elongated hypocotyls and closed, yellowish cotyledons. In contrast, exposure to light, particularly at optimal temperatures (i.e. 22 °C for Arabidopsis), initiates photomorphogenesis, resulting in shorter hypocotyls and open, green cotyledons (Osterlund et al. 2000). Plant morphological and architectural adaptations in response to warm temperatures are known as thermomorphogenesis. These changes include significantly elongated roots, hypocotyls, petioles, and early flowering phenotypes (Casal and Balasubramanian 2019). Notably, light and temperature antagonistically regulate hypocotyl growth. While light inhibits hypocotyl elongation, warm temperatures promote elongation (Bian et al. 2022).

Photoreceptors, including phytochromes, cryptochromes, phototropins, and UVR8, play pivotal roles in perceiving various light signals and transmitting them to downstream signaling components to precisely regulate plant growth and development (Paik and Huq 2019). Phytochrome B (phyB) is a critical player capable of integrating light and temperature signals (Chen et al. 2022). Upon activation by red light, phyB converts to the active Pfr form, which facilitates the degradation of PHYTOCHROME INTERACTING FACTORs (PIFs). This process promotes photomorphogenesis, particularly at optimal growth temperatures by the activation of positive regulators of photomorphogenesis. Conversely, in dark or far-red light conditions or under warm temperatures, phyB shifts to its inactive Pr state, allowing the accumulation of PIFs (Bian et al. 2022). Of these, PIF4 is of particular interest as it acts as a central hub, integrating signals from light and temperature cues to regulate plant growth and development (Koini et al. 2009).

The molecular networks are often cross-linked with several endogenous hormones. Recent studies have shed light on the involvement of jasmonic acid (JA) signaling in integrating light and temperature responses. JA represses hypocotyl growth and promotes cotyledon unfolding in dark-grown Arabidopsis seedlings by inhibiting skotomorphogenesis genes. This repression is mediated by the ubiquitination and degradation of JASMONATE ZIM-DOMAIN (JAZ) proteins, key repressors of JA signaling (Zheng et al. 2017). Warm temperatures induce the expression of JA catabolism genes, promoting hypocotyl elongation in Arabidopsis. External application of JA can de-repress warm temperature–mediated hypocotyl elongation (Zhu et al. 2021). However, the specific components of JA signaling that connect light and temperature cues remain to be fully elucidated.

In this issue of *Plant Physiology*, Huai and co-workers (Huai et al. 2024) investigated a molecular mechanism by which JA signaling integrates light and temperature responses in shaping seedling morphogenesis in Arabidopsis. Among 13 *JAZ* genes in Arabidopsis, the authors found that overexpression of *JAZ3* (*35S:JAZ3-GFP*) resulted in notable phenotypic changes such as shorter hypocotyls and cotyledon unfolding under various light conditions (continuous red, far-red, blue, and low-intensity white light conditions). However, phenotypes of *JAZ3-GFP* and *jaz3* mutant lines did not differ from wild type in the dark. Furthermore, the study revealed that overexpression of *JAZ3* inhibited warm temperature–mediated hypocotyl elongation at 28 °C, underscoring its role in thermomorphogenesis as well. This dual regulation of seedling morphogenesis by JAZ3 suggests its significance in coordinating responses to light and temperature cues.

Huai et al. (2024) observed that the JAZ3 protein formed dynamic nuclear speckles in *Nicotiana bethamiana* leaves. Using fluorescence recovery after photobleaching (FRAP), the authors confirmed that JAZ3 proteins undergo liquid-liquid phase separation (LLPS), a process facilitated by its prion-like low complexity domain (PrD), which is unique to JAZ3 among the JAZ proteins. Importantly, Huai et al. (2024) demonstrated the interaction between JAZ3 and the transcription factor PIF4, which is known to integrate light and temperature signals. While PIF4 alone does not undergo LLPS due to the absence of a typical PrD, co-expression of JAZ-GFP and PIF-mCherry promoted the LLPS of PIF4. Sequestration of PIF4 into liquid droplets by JAZ3 inhibits the DNA binding ability of PIF4 and thus represses the transcriptional activation of its target genes such as *EXPANSIN2*, *INDOLE-3-ACETIC ACID INDUCIBLE 19*, and *YUCCA8* (Fig.).

**Figure.**
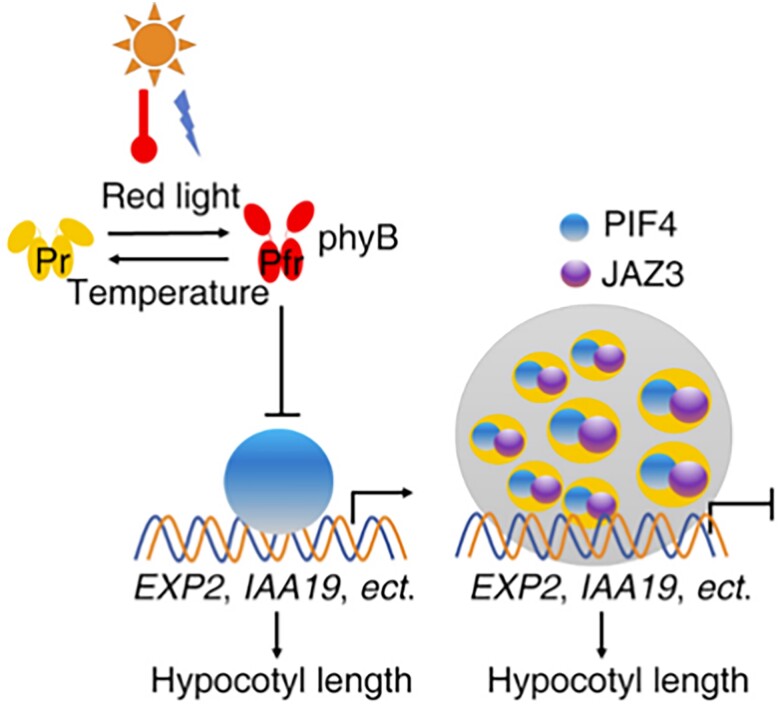
Proposed model of how JAZ3 mediates light and temperature cues. Adapted from Huai et al. (2024). In various light conditions and warm temperatures, JAZ3 overexpression results in the sequestration of PIF4 into liquid droplets and thereby represses PIF4-mediated hypocotyl elongation.

In summary, this study uncovers the crucial role of JAZ3 in integrating light and temperature signaling pathways in Arabidopsis seedling morphogenesis. By interacting with PIF4 and promoting its phase separation, JAZ3 acts as a critical regulator, modulating PIF4-mediated responses, such as hypocotyl elongation and cotyledon expansion (Fig.). Future research endeavors may unravel the functional redundancy of JAZ proteins, as evidenced by the lack of JA-mediated photo and thermo-morphogenic phenotypes in the *jaz3* mutant line under dark and warm temperatures. Moreover, the potential existence of functional redundancy among JAZs raises intriguing questions about the diverse molecular mechanisms through which these proteins regulate responses to light and temperature cues, particularly given that JAZ3 exclusively contains the PrD domain. Understanding the complex regulatory mechanisms governing plant responses to light and temperature is crucial for deciphering how plants adapt to changing environmental conditions. It also informs the development of strategies to enhance crop productivity and resilience in the face of climate change.

## Dive Curated Terms

The following phenotypic, genotypic, and functional terms are of significance to the work described in this paper:

PIF4 Gramene: AT2G43010
PIF4 Araport: AT2G43010

UVR8 Gramene: AT5G63860
UVR8 Araport: AT5G63860

JAZ3 Gramene: AT3G17860
JAZ3 Araport: AT3G17860
